# Acknowledgement to Reviewers 2023

**DOI:** 10.3389/ijph.2024.1607100

**Published:** 2024-01-31

**Authors:** IJPH Editorial Office

**Affiliations:** Swiss School of Public Health (SSPH+), Zürich, Switzerland

The Editors of the *International Journal of Public Health* would like to thank all Reviewers in 2023 for the time they have spent and the valuable contributions they have made to ensure the scientific quality of the journal.

Charles Abongomera, Switzerland

Karim Abu-Omar, Germany

Laura Acosta, Argentina

Magdalena Adamus, Slovakia

Adekunle Adedeji, Germany

Claudia Agnoli, Italy

Junaid Ahmad, Pakistan

Zaher Ahmad Nazzal, Palestine

Mueen Ahmed, India

Ahsan Ahsan, Indonesia

Fatme Al Anouti, United Arab Emirates

Karim Al-Jashamy, Malaysia

Anmar Al-Taie, Turkey

Anokhi Ali Khan, Switzerland

Regina Allande Cussó, Spain

Asrar Alobaidy, Iraq

Francisco Alonso, Spain

Rebecca Amati, Switzerland

Heresh Amini, United States

Desalegn Amsalu, Ethiopia

Ankit Anand, India

Mirko Ancillotti, Sweden

Giovanni Annuzzi, Italy

Jon Anson, Israel

Thomas Arcury, United States

Atsede Aregay, Norway

Alex Ario, Uganda

Richard Arnold, New Zeland

Shahram Arsang-Jang, United States

Deodato Assanelli, Italy

Muhammad Atiq, Pakistan

Zeynep Meva Altaş, Turkey

Abolfazl Avan, Canada

Sina Azadnajafabad, Iran

Noshaba Aziz, China

Maria Bacikova-Sleskova, Slovakia

Georgian Badicu, Romania

Mahnaz Badpa, Germany

Rahim Badrfam, Iran

Yu Bai, China

Peter Bakalár, Slovakia

Se-Sergio M. Baldew, Suriname

Joseph Balikuddembe, China

Kiran Bam, Australia

Delia Bancila, United States

Caroline Barakat, Canada

Franca Barbic, Italy

Cristina Barboza-Solis, Costa Rica

Emmanuel Baron, France

Peter Barrett, Ireland

Friederike Barthels, Germany

Tibor Baska, Slovakia

Jorgen Bauwens, Switzerland

Tevfik Bayram, Canada

Eduardo Becerril, Mexico

Chance Bell, United States

Finaba Berete, Belgium

Lisa Berg, Sweden

Bijit Biswas, India

Uwe H. Bittlingmayer, Germany

Zuzana Boberová, Slovakia

Giovanna Boccuzzo, Italy

Marialaura Bonaccio, Italy

Alberto Borraccino, Italy

Frederic Bouder, Norway

Brett Bowman, South Africa

Jadranka Bozikov, Croatia

Elise Braekman, Belgium

Andreja Brajsa Zganec, Croatia

Shuli Brammli-Greenberg, Israel

Tilman Brand, Germany

Nurlan Brimkulov, Kyrgyzstan

Donka Brodersen, United States

Sergiy Bronin, Ukraine

Heather Brown, United Kingdom

Leticia Brown, United States

Helena Bruggeman, Belgium

Michał Brzeziński, Poland

Alexandre Bulgarelli, Brazil

Chris Buse, Canada

Luis Calmeiro, Singapore

Dan Cao, China

Mabel Carabali, Canada

Claudia Cardone, Italy

Santiago Carranco, Ecuador

Raphaële Castagné, France

Alberto Castro Fernandez, Switzerland

Alessandro Ceschi, Switzerland

Duncan Chambers, United Kingdom

Jie Chen, China

Pei-Ching Chen, Taiwan

Ruoke Chen, Canada

Yun Chen, China

Afona Chernet, Switzerland

Francesco Chirico, Italy

Luiz Alexandre Chisini, Brazil

Patricia Chocano-Bedoya, Switzerland

Marco Ciccone, Italy

Volker Cihlar, Germany

Aaron Cohen, United States

Yates Coley, United States

Andrea Conti, Italy

Alessandra Costanza, Switzerland

Simona Costanzo, Italy

Sara Cottler-Casanova, Switzerland

Gaoussou Coulibaly, Côte d'Ivoire

Stéphane Cullati, Switzerland

William Dab, France

Gavin Dabrera, United Kingdom

Paola Daniore, Switzerland

Zuzana Dankulincová, Slovakia

Rita De Siano, Italy

Claire Deacon, South Africa

Anne Dee, Ireland

Paola Delbon, Italy

Peter Delobelle, South Africa

Ali Delshad Noghabi, Iran

Daniel Demant, Australia

Bahadir Demir, Turkey

Evangelia Demou, United Kingdom

Domenico Depalo, Italy

Augusto Di Castelnuovo, Italy

Giuseppe Di Martino, Italy

Laura Di Giorgio, United States

Micaela Di Consiglio, Italy

Gian Luca Di Tanna, Switzerland

Patricia Dias, Brazil

Julio Diaz, Spain

Hassan H. Dib, Canada

Helga Dizdari, Switzerland

Shirin Djalalinia, Iran

Martin Dlouhy, Czechia

Maria Do Rosário, Portugal

Dana Dolanová, Czechia

Maria Felicitas Dominguez-Berjon, Spain

Shen Dong, China

Keren Dopelt, Israel

Jyoshma Dsouza, France

Simon Ducarroz, France

Tamer Edirne, Turkey

Tara Elton-Marshall, Canada

Carla Enes, Brazil

Stefan Essig, Switzerland

Ugochukwu Anthony Eze, Nigeria

Marta Fadda, Switzerland

Irene Falgas Bagu, Switzerland

Ya Fang, China

Pai Feng Hsu, Taiwan

Paolo Ferrari, Switzerland

Augusto César Ferreira De Moraes, United States

Melissa Fiffer, United States

Massimo Finocchiaro Castro, Italy

Maddalena Fiordelli, Switzerland

Igor Francetic, United Kingdom

Anja Frei, Switzerland

Ana Freitas Ribeiro, Brazil

Benjamin Frey, Germany

Adriano Friganović, Croatia

Rajendra Gadhav, India

Udo S. Gaipl, Germany

Artur Galimov, United States

Tais Galvao, Brazil

Manuel García-Ramírez, Spain

Antonio Garofalo, Italy

Barbara Gärtner, Germany

Teresa Gavaruzzi, Italy

Leon Geffen, South Africa

Karin Geffert, Germany

Jana Gerold, Switzerland

Gebrenegus Ghilagaber, Sweden

Ahona Ghosh, India

Santu Ghosh, India

Anwal Ghulam, Italy

Francesco Gianfagna, IItaly

Emilio Gianicolo, Germany

Ingrid Gilles, Switzerland

Thaisa Gois Farias de Moura Santos Lima, Brazil

Dulce Gomes, Portugal

Juan Gómez-Salgado, Spain

Paulo Goncalves, Switzerland

Judite Gonçalves, United Kingdom

Neus González, Spain

Jose Luis González-Castro, Spain

Adrián González Marrón, Spain

Patrick Goodman, Ireland

Mark A. Gottlieb, United States

Margaret Gough Courtney, United States

Ian Grant, United Kingdom

Lennert Griese, Germany

Leonor Guariguata, Belgium

Negnorogo Guindo-Coulibaly, Côte d'Ivoire

Sarah R. Haile, Switzerland

Zdenek Hamrik, Czechia

Bing Han, China

Ziqiang Han, China

Reiner Hanewinkel, Germany

Stephen Hanney, United Kingdom

Otto Hänninen, Finland

Mohammad Adrian Hasdianda, United States

Jaime Hart, United States

Iris Hartog, Netherlands

Margaret Haworth-Brockman, Canada

Bin He, China

Frank Heiland, United States

Urs Hepp, Switzerland

Lize Hermans, Belgium

Pablo Hernández, México

Uy Hoang, United Kingdom

Alice Hocquette, France

Paul Hogan, United States

Jian Hou, China

Lei Hou, China

Silva Hovsepian, Iran

Cheng-Yang Hu, China

Yu-Te Huang, China

Chia Hui Tan, Taiwan

Isabel Hurtado, Colombia

Daniela Husarova, Slovakia

Maria Isabel Gutierrez, Colombia

Michał Jacek Jędrzejek, Poland

Kristen Jafflin, Switzerland

Sara JalaliFarahani, United Kingdom

Slobodan Jankovic, Serbia

Christian Janssen, Germany

Alvaro Javier Idrovo, Spain

Ayan Jha, United States

Anne Jolivet, France

Mogan Ka, India

Maria Kamenetsky, United States

Peter Kamstra, Australia

Ozlem Kar Kurt, Turkey

Emir Karacaglar, Turkey

Abhradeep Karmakar, United States

Lum Kastrati, Switzerland

Harpriya Kaur, United States

Ilker Kayi, Turkey

Sherrie Kelly, Switzerland

Anna Kemp-Casey, Australia

Michal Kentos, Slovakia

Pauline Kergall, France

Şiran Keske, Turkey

Alireza Khajavi, Iran

Ibrahim Khali, United States

Nazeer Hussain Khan, United States

Meenakshi Khapre, India

Khom Raj Kharel, Nepal

Farnaz Khatami, Switzerland

Seblewongiel Kidanie, Ethiopia

Judith Kimiywe, Kenya

Nikolai Kiselev, Switzerland

Brandon Knettel, United States

Pablo Knobel, United States

Philipp Kohler, Switzerland

Ulrich Kohler, Germany

Leena Koivusilta, Finland

Veronika Korim, Slovakia

Michaela Kosticova, Slovakia

Eleni Koutsogeorgou-Andreou, United Kingdom

Sari Kovats, United Kingdom

Eva Kralikova, Czechia

Min Kuang Tsai, Taiwan

Yuta Kubo, Japan

Nino Kuenzli, Switzerland

Yuli Kusumawati, Indonesia

Kerem Laçiner, Turkey

Samanta Lalla-Edward, South Africa

Tyler Lane, Australia

Carlos Laranjeira, Portugal

Guy Launoy, France

Victoria Leclercq, Belgium

Zohar Lederman, China

Hyo Lee, Republic of Korea

Michela Lenzi, Italy

Žan Lep, Slovenia

Gobopamang Letamo, Botswana

Rosamund F. Lewis, Switzerland

Katarzyna Lewtak, Poland

Binqi Li, China

Wenyuan Li, China

Crystal Lim, United States

Marta Lima-Serrano, Spain

Chien-Yu Lin, Japan

Vaclav Linkov, Slovakia

Elena Lisá, Slovakia

Zhonghua Liu, United States

Giuseppina Lo Moro, Italy

Steffen Loft, Denmark

Pablo Lopez, Argentina

Sergio López García, Spain

Sandrine Loubiere, France

Louis Loutan, Switzerland

Yihan Lu, China

Daniel Ludecke, Germany

Yuxi Luo, China

Leigh M. McClarty, Canada

Katie MacLure, United Kingdom

Luiz Fernando Machado, Brazil

Andrea Madarasova Geckova, Slovakia

Kalana Prasad Maduwage, Sri Lanka

Anselimo Makokha, Kenya

Tatjana Makovski, France

Christos Andreas Makridis, United States

Fred Maniragaba, Uganda

Erma Manoncourt, France

Tsegahun Manyazewal, Ethiopia

Rafael Manzanera, Spain

Zhenxing Mao, China

Mardiana Mardiana, Indonesia

Lucia Maria Lotrean, Romania

Jimmy Martin-Delgado, Ecuador

Claudia Martínez, Italy

María Ángeles Martínez, Spain

Dmytro Martsenkovskyi, Ukraine

Rainier D. Masa, United States

Erika Maskálová, Slovakia

Darja Maslić Seršić, Croatia

Balcha Masresha, Republic of Congo

Juergen Maurer, Switzerland

Joanna Mazur, Poland

David McQueen, Switzerland

Roberto Mediavilla, Spain

Christos Melidis, France

Fabian Mendez, Colombia

Brenna Menke, United States

Rowena Katherine Merritt, United Kingdom

Sonja Merten, Switzerland

Sven Messing, Germany

Takehiro Michikawa, Japan

Jelena Milic, Serbia

Philipa Mladovsky, United Kingdom

Christian Monsalve, United States

Matthis Morgenstern, Germany

Janeth Mosquera Becerra, Colombia

Mildrey Mosquera, Colombia

Gregory Moullec, Canada

Ujjaini Mukhopadhyay, India

Charles Muriuri, United States

Joanna Myszkowska-Ryciak, Poland

Nicolette Nabukeera-Barungi, Uganda

Mehrdad Naderi, Taiwan

Etheldreda Nakimuli-Mpungu, Uganda

Damalie Nalwanga, Uganda

Rebecca Nantanda, Uganda

Rubén Navarro-Patón, Spain

Nawi Ng, Sweden

Nhung Nguyen, Vietnam

Thang Nguyen-Tien, Kenya

Kathryn Nicholson, Canada

Helene Niculita-Hirzel, Switzerland

Rajen Nithiseelan Naidoo, South Africa

JIraluck Nontarak, Thailand

Jennifer O'Loughlin, Canada

Katrina Obas, Switzerland

Kathryn Oliver, United Kingdom

Doug Oman, United States

Stephen Okumu Ombere, Kenya

Elijah Onsomu, United States

Simeon Onyemaechi, Nigeria

Meryem Merve Oren, Turkey

Michel Oris, Switzerland

Secil Ozkan, Turkey

Veronika Pacutova, Slovakia

Stefania Paduano, Italy

Antonio Palacios-Rodríguez, Spain

Lucia Palandri, Italy

Raffaele Palladino, Italy

Salvatore Panico, Italy

Roberta Parisi, Italy

Daria Pašalić, Croatia

Miguel Peralta, Portugal

Cuong Pham, Vietnam

William Pickett, Canada

Christoph Pimmer, Switzerland

Betine Pinto Moehlecke Iser, Brazil

Carla Pires, Portugal

Cristina Bianca Pocol, Romania

Veruska Prado Alexandre Weiss, Brazil

Patrick Präg, France

Daicia Price, United States

Michele Provenzano, Italy

Mohammad Habibullah Pulok, Canada

Parul Puri, India

Mauricio Quintero, Colombia

Oliver Rácz, Slovakia

Athina Raftopoulou, Greece

Ossi Rahkonen, Finland

Viviane Ramel, France

Pilar Ramos, Spain

Muhammad Adil Rasheed, Pakistan

Atta Rehman, Pakistan

Eva Rens, Belgium

Zahra Reynolds, United States

Fulvio Ricceri, Italy

Susan Rifkin, United States

Zayne Roa, Switzerland

Tony Robertson, United Kingdom

Jean-Marie Robine, France

Daniela Rodrigues, Portugal

Ángel Rodríguez Laso, Spain

Domenica Romeo, Italy

Aldo Rosano, Italy

Kathryn Rose, Australia

Arianna Rotulo, Netherlands

Alessandro Rovetta, Italy

Adriana Rusu, Romania

Pier Luigi Sacco, Italy

Jihene Sahli, Tunisia

Hanen Samouda, Luxembourg

Jamile Sanches Codogno, Brazil

Teresa Santos, Portugal

Abdullah Sarıöz, Turkey

Apolline Saucy, Spain

Marie-Josephe Saurel-Cubizolles, France

Mustafa Saygin, Turkey

Cono Scaffa, Italy

Simone Schenkman, Brazil

Diana Schow, United States

Bettina Schwind, Switzerland

Jamie Seabrook, Canada

Maureen Seguin, United Kingdom

Maninder Setia, India

Anna Ševčíková, Czechia

Tang Shangfeng, China

Stav Shapira, Israel

Martin Sherman, United States

Akira Shibanuma, Japan

Wondimeneh Shiferaw, Ethiopia

Altynay Shigayeva, Canada

Edward Shiu, United Kingdom

Dagmar Sigmundová, Czechia

Carlo Signorelli, Italy

Jane Silovsky, United States

Vittorio Simeon, Italy

Jean Simos, Switzerland

Geetu Singh, India

Marko Siroglavić, Croatia

Zuzana Skodova, Slovakia

Jeff Slezak, United States

Patrícia Soares, Portugal

Natasha Sobers, Barbados

Biju Soman, India

Sajid Soofi, Pakistan

Amandia Sousa, Brazil

Cristiane Spadacio, Brazil

Stefanie Sperlich, Germany

Giovanni Spitale, Switzerland

Chandrashekhar Sreeramareddy, Malaysia

Christiane Stock, Germany

Jenna Strizzi, Denmark

Gorden Sudeck, Germany

Jiahong Sun, China

Sakari Suominen, Finland

Dewi Susanna, Indonesia

Paula Tallman, United States

Wendy K. Tam, United States

Babar Tasneem Shaikh, Pakistan

William Tebar, Brazil

Sidika Tekeli-Yesil, Switzerland

Tassia Teles Santana De Macedo, Brazil

Jean Tenena Coulibaly, Côte d'Ivoire

Annemarie Ter Veen, Switzerland

Gudina Terefe Tucho, Ethiopia

Zelalem Terfa, United Kingdom

Louis Thiemann, Germany

Sarina Till, South Africa

Elaine Tomasi, Brazil

Tatiana Toporcov, Brazil

Philipp Trein, Switzerland

Maurizio Trevisan, Vietnam

Christopher Troeger, United States

Athanasios Tsaraklis, Greece

Charalampos Tsavdaroglou, Greece

Shinya Tsuzuki, Japan

Sergii Tukaiev, Ukraine

Serhii Tukaiev, Switzerland

Yasin Tülüce, Turkey

Wenceslao Unanue, Chile

Dipak Prasad Upadhyaya, United States

Maja Vajagic, Croatia

Patricio Valenzuela, Chile

Nerida Nadia H. Valero, Brazil

Antonio Vallinoto, Brazil

Antje Van Der Zee-Neuen, Austria

Jeroen Van Der Waal, Netherlands

Johan Van Der Heyden, Belgium

Piet Van Eeuwijk, Switzerland

Raf Van Gestel, Netherlands

Louella Vaughan, United Kingdom

Paolo Miguel Manalang Vicerra, China

Francesco Vidoli, Italy

Anne Vinkel Hansen, Denmark

Aleksandar Višnjić, Serbia

Matteo Vitali, Italy

David Vizcaya, Germany

Peter Von Philipsborn, Germany

Viktor von Wyl, Switzerland

Isidora Vujcic, Serbia

Jordyn Wallenborn, Switzerland

Mkhululi Wami, Netherlands

Anqi Wang, United States

Qing Wang, China

Shaobin Wang, United States

Shengnan Wang, China

Siquan Wang, United States

Xinpei Wang, China

Xuemei Wang, China

Grace Waweru, Kenya

Yehua Wei, United States

James West, United States

Michael White, United States

Benedikt Wicki, Switzerland

Ander Wilson, United States

Caradee Wright, South Africa

Xizhu Xiao, China

Neşe Yakşi, Turkey

Xue Yan, China

Fan Yang, China

Shujuan Yang, Iran

Tianan Yang, China

Aisha Yansaneh, United States

Kae Yi Tan, Malaysia

Ferhat Yildiz, Turkey

Xinwen Yu, China

Yongfu Yu, China

Denis Yukhnenko, United Kingdom

Renee Zahnow, Australia

Virginia Zarulli, Denmark

Aurel Zelko, Slovakia

Nejimu Zepro, Switzerland

Ivan Žežula, Slovakia

Jiawei Zhang, Denmark

Yaohui Zhao, China

Luis Zingg, Netherlands

